# Die „Fit im Nordwesten“-Toolbox: Entwicklung und Bereitstellung von Materialien und Empfehlungen zur Förderung körperlicher Aktivität älterer Menschen im kommunalen Setting

**DOI:** 10.1007/s11553-021-00926-w

**Published:** 2021-12-06

**Authors:** Manuela Peters, Tiara Ratz, Saskia Muellmann, Jochen Meyer, Kai von Holdt, Claudia Voelcker-Rehage, Sonia Lippke, Claudia R. Pischke

**Affiliations:** 1 Leibniz-Institut für Präventionsforschung und Epidemiologie – BIPS, Bremen, Deutschland; 2 Jacobs University Bremen, Bremen, Deutschland; 3 OFFIS – Institut für Informatik, Oldenburg, Deutschland; 4 Institut für Sportwissenschaft, Westfälische Wilhelms-Universität Münster, Münster, Deutschland; 5 Institut für Medizinische Soziologie, Centre for Health and Society, Medizinische Fakultät, Heinrich-Heine-Universität Düsseldorf, Düsseldorf, Deutschland

**Keywords:** Bewegungsintervention, Gesundheitsförderung, Gesundes Altern, Praxismaterialien, Primärprävention, Exercise intervention, Health promotion, Healthy aging, Practice materials, Primary prevention

## Abstract

**Hintergrund:**

Regelmäßige körperliche Aktivität ist von zentraler Bedeutung für gesundes Altern. Allerdings halten in Deutschland weniger als ein Viertel der ab 65-Jährigen die Aktivitätsempfehlungen der Weltgesundheitsorganisation ein.

**Ziel der Arbeit:**

In zwei Studienphasen (PROMOTE I und II) wurden web- und print-basierte Programme zur Förderung körperlicher Aktivität älterer Erwachsener ab 60 Jahren entwickelt und mittels randomisierter Interventionsstudien analysiert und evaluiert. Ziel dieses Beitrags ist es, die daraus resultierenden Empfehlungen und Materialien, die im Rahmen einer Toolbox für Anbieter:innen von Bewegungsprogrammen im kommunalen Setting angeboten werden, vorzustellen.

**Material und Methoden:**

Insgesamt erhielten 651 Personen über 10 Wochen Zugang zu der web- oder print-basierten „Fit im Nordwesten“-Bewegungsintervention. In der zweiten Studienphase (PROMOTE II) wurde das Programm mithilfe eines partizipativen Ansatzes an bisher inaktive Ältere angepasst. Die Zufriedenheit mit der Intervention und die Effektivität hinsichtlich des Bewegungsverhaltens wurden wissenschaftlich evaluiert.

**Ergebnisse:**

Die „Fit im Nordwesten“-Toolbox umfasst theoriebasierte, praxisrelevante und wissenschaftlich evaluierte Handlungsempfehlungen für die Förderung der körperlichen Aktivität älterer Erwachsener ab 60 Jahren. Die bereitgestellten Dokumentensets enthalten Materialien für 1) die Vor- und Nachbereitung, 2) die Durchführung eines zehnwöchigen Bewegungsprogramms, 3) die langfristige Aufrechterhaltung des Bewegungsverhaltens und 4) die Begleitung für Theorie und Praxis.

**Schlussfolgerung:**

Bisher existierten kaum wissenschaftlich evaluierte Materialien für die praktische Nutzung in der Bewegungsförderung. Die „Fit im Nordwesten“-Toolbox kann die zukünftige Anwendung in Bewegungsprogrammen für ältere Erwachsene unterstützen.

## Abstract (en)

**Background:**

Regular physical activity is central to healthy ageing. However, less than 25% of German older adults aged 65 years or older currently reach the World Health Organization’s recommendations.

**Objectives:**

In two study phases (PROMOTE I and II), web- and print-based programs to promote physical activity in older adults aged 60 years and older were developed. Intervention effectiveness was evaluated in randomized intervention trials. The goal is to present the recommendations and materials that emerged and were summarized in a toolbox for use in intervention planning and implementation in the community setting.

**Materials and methods:**

A total of 651 individuals received access to the web- or print-based “Fit im Nordwesten” physical activity intervention for 10 weeks. As part of the PROMOTE II study, the program was adapted to initially inactive older adults using a participatory approach. Acceptance and intervention effectiveness regarding physical activity behavior were scientifically evaluated.

**Results:**

The “Fit im Nordwesten” toolbox includes theory-based, practice-relevant, and scientifically evaluated recommendations for the promotion of physical activity among older adults aged 60 years and older. The provided document sets include materials for (1) the preparation and postprocessing, (2) the implementation of the 10-week physical activity program, (3) supporting long-term maintenance of physical activity behavior, and (4) supporting materials for theory and practice.

**Conclusions:**

Thus far, the provision of scientifically evaluated materials for practical use in the promotion of physical activity was limited. The findings and recommendations presented here as part of the “Fit im Nordwesten” toolbox can support future application in physical activity programs targeting older adults.

Im Rahmen der Förderung gesunden Alterns ist regelmäßige Bewegung ein zentraler Baustein. In Deutschland bewegt sich mehr als die Hälfte der älteren Erwachsenen nicht ausreichend. Strukturierte und evidenzbasierte Bewegungsangebote sind notwendig, um Menschen ab 60 Jahren dabei zu unterstützen, körperlich aktiv zu werden und langfristig zu bleiben. Trotz umfassender Forschung zur Effektivität von Bewegungsinterventionen mangelt es an der Bereitstellung wissenschaftlich evaluierter Materialien, die zu einer theorie- und evidenzbasierten Nutzung in der Praxis befähigen.

## Einleitung

### Hintergrund

Beinahe die Hälfte der deutschen Bevölkerung wird in Zukunft über 60 Jahre alt sein [1]. In Anbetracht dieser erwarteten Entwicklung ist es von Bedeutung, die Gesundheit und Lebensqualität dieser Bevölkerungsgruppe zu erhalten bzw. zu stärken. Dazu gehört Krankheitsprävention ebenso wie Gesundheitsförderung. Insbesondere regelmäßiger körperlicher Aktivität wird ein enormes Potenzial zugeschrieben, Gesundheit und Wohlbefinden Älterer zu erhalten bzw. zu steigern [4, 5, 15, 17]. In diesem Zusammenhang empfiehlt die Weltgesundheitsorganisation (WHO) Erwachsenen ab 65 Jahren ein moderates bis intensives Ausdauertraining von mindestens 150 min pro Woche sowie ein zusätzliches Beweglichkeits‑, Kraft- und Gleichgewichtstraining [18, 19]. Da allerdings weniger als ein Viertel der älteren Erwachsenen in Deutschland diese Empfehlungen erfüllt [2], sind Bewegungsangebote, die die individuellen Leistungsniveaus der Zielgruppe berücksichtigen und so gestaltet sind, dass diese motiviert bleibt, essenziell. Während für viele jüngere Menschen die Leistungssteigerung, das Siegen oder Erleben extremer Situationen beim Sport bedeutsam sind, gewinnen für Menschen höheren Alters die Themenfelder Gesundheit, Fitness und Wohlbefinden an Bedeutung [16]. In diesem Zusammenhang ist es sinnvoll, Angebote auf diese Themenfelder und Motivationshintergründe auszurichten. Diese könnten auch bislang inaktiven älteren Erwachsenen dabei helfen, regelmäßig aktiv zu werden und möglichst zu bleiben.

Evidenzbasierte Angebote können (print- oder web-basierte) Übungs- und Informationsmaterialien [7–9], aber auch angeleitete Bewegungseinheiten oder ganze Programme beinhalten, die vor Ort mit der Zielgruppe oder zu Hause durchgeführt werden können. Auch ein persönliches Bewegungsmonitoring, z. B. anhand von Bewegungstagebüchern [6], kann ein hilfreiches Tool für die Zielgruppe sein, sich das persönliche Bewegungsverhalten zu vergegenwärtigen und das Niveau graduell zu steigern. Im Rahmen der PROMOTE-Studie (mit den Studienphasen I und II) wurden derartige Tools, d. h. web- oder printbasierte Materialien auf ihre Eignung zur Bewegungsförderung bei inaktiven älteren Erwachsenen wissenschaftlich untersucht. Manuskripte mit Ergebnissen hierzu befinden sich derzeit noch in der Begutachtung [11, 14].

### Die PROMOTE-Studie

Die PROMOTE-Studie (Projektstart im Jahr 2015) ist ein Teilprojekt des regionalen Präventionsforschungsnetzwerks „Körperliche Aktivität, Gerechtigkeit und Gesundheit: Primärprävention für gesundes Altern“ (AEQUIPA [3]). Das definierte, übergeordnete Ziel ist es, theoriebasierte und empirische Interventionen zur verhältnis- und verhaltensbasierten Bewegungsförderung für Erwachsene ab 65 Jahren zu entwickeln und zu evaluieren. Dabei stehen insbesondere der Einsatz neuer Technologien (z. B. digitale Komponenten) sowie partizipative Ansätze im Vordergrund. Letztere sollen die interdisziplinäre Zusammenarbeit der angewandten Forschung und Entwicklung mit der Praxis stärken [6]. Die Dissemination und Implementation der Forschungsergebnisse an/für die interessierte Öffentlichkeit und politische Entscheidungsträger:innen sind hierbei bedeutsame Teilziele.

Im Zuge der Evaluation und Analyse der Ergebnisse der ersten Studienphase (PROMOTE I) deuteten sich bereits Verbesserungen hinsichtlich der körperlichen Aktivität an [9]. So konnte das Bewegungsprogramm zu einer Steigerung der Selbstwirksamkeit, der individuellen Intention und Planungsfähigkeit für das empfohlene Bewegungsverhalten führen [13]. Analysen aus der zweiten Studienphase weisen ebenfalls z. T. auf positive Effekte hin [11, 14]. Im Rahmen der Dissemination werden die auf dieser wissenschaftlichen Grundlage fußenden Erkenntnisse nun als Materialien für Akteur:innen, die in die Programmplanung und -durchführung von Bewegungsangeboten für ältere Erwachsene involviert sind, als Toolbox bereitgestellt.

### Studienziel

Ziel von PROMOTE war die Entwicklung web- und print-basierter Interventionen zur Förderung körperlicher Aktivität älterer Erwachsener ab 60 Jahren auf Basis randomisierter Interventionsstudien, deren Evaluation und Dissemination. Ein Schwerpunkt der Bewegungsintervention war die Erarbeitung von Maßnahmen zur Unterstützung der Selbstregulation und einer langfristigen Gewohnheitsbildung in Bezug auf einen körperlich aktiven Lebensstil. Detaillierte Hintergründe zu PROMOTE wurden bereits an anderer Stelle berichtet [8, 10].

Ziel dieses Beitrags ist die Beschreibung der in Form einer „Toolbox“ bereitgestellten Materialien und Empfehlungen für den praktischen Einsatz in der Prävention und Gesundheitsförderung.

## Methoden

### Partizipative Entwicklung der Materialien

**Abb. 1:**
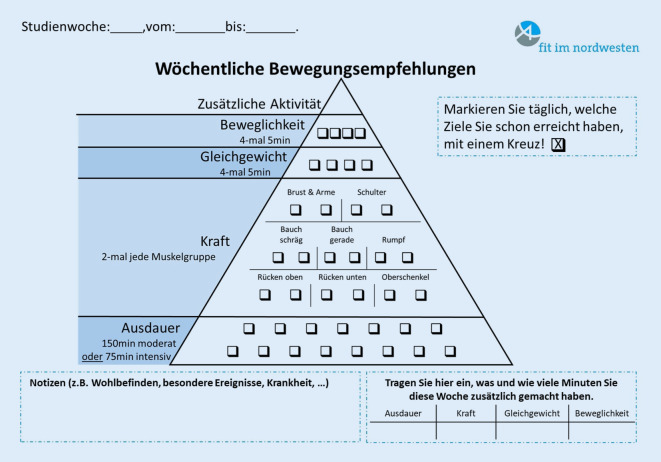
Bewegungspyramide aus der PROMOTE-II-Studie

Da die in die erste Studienphase (PROMOTE I) eingeschlossenen Teilnehmer:innen ein bereits verhältnismäßig hohes Aktivitätslevel aufwiesen, wurden als Zielgruppe für die zweite Studienphase (PROMOTE II) weitgehend inaktive ältere Erwachsene im kommunalen Setting (Wohnumfeld, Gemeinde, Stadt) definiert [10]. Daraus resultierte eine grundlegende Überarbeitung der Zielgruppenansprache als auch der Interventionsmaterialien. Dies inkludiert die Adaption der web-basierten Materialen sowie die Entwicklung eines print-basierten Bewegungstagebuchs für Personen mit geringer ausgeprägter Technikaffinität (Abb. 1).

**Abb. 2:**
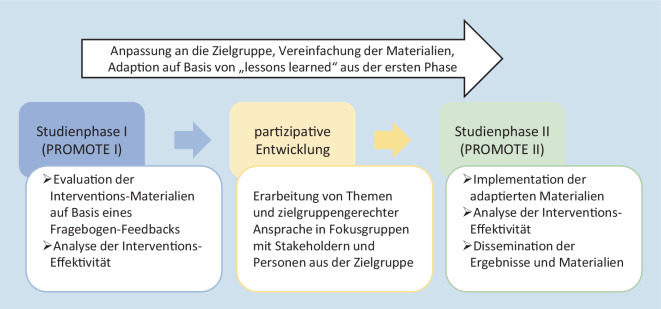
Adaption der Interventionsmaterialen zwischen zwei Studienphasen

Um die in PROMOTE I evaluierten web-basierten Komponenten des „Fit im Nordwesten“-Bewegungsprogramms in eine print-basierte Version zu überführen, wurden in der Interventionsentwicklungsphase von PROMOTE II vom Studienpersonal geleitete Fokusgruppen mit *n* = 12 Stakeholdern (z. B. Seniorenbeiratsmitglieder, Ortsamtsleitende) und *n* = 32 Personen aus der Zielgruppe (nicht ausreichend körperlich aktive ältere Erwachsene) durchgeführt (Abb. 2 und Materialen im frei zugänglichen Projekt-Repository [12]). Die Veranstaltungen dienten der Identifikation der Bedürfnisse und Wünsche bisher nicht regelmäßig körperlich aktiver älterer Erwachsener. In diesem Kontext wurden Materialien – sowohl print-basierte Komponenten, die bereits in PROMOTE genutzt wurden, als auch diverse Broschüren von Krankenkassen, dem Bundesministerium für Gesundheit, der Bundeszentrale für gesundheitliche Aufklärung oder der Deutschen Gesellschaft für Ernährung (mit Informationen, Tipps, Übungen, Selbsttests und Hilfsmitteln zur Unterstützung eines gesunden Lebensstils) – hinsichtlich ihrer Eignung für das Studienziel und die Zielgruppe diskutiert. Unter Einsatz der didaktischen Methode des *World Cafés* (s. Infobox 1) wurden in Kleingruppen Fragen zu wesentlichen Aspekten gesunden Alterns; Möglichkeiten der Erreichung der Zielgruppe (inaktive ältere Erwachsene) sowie Motivationsfaktoren zur Aufrechterhaltung eines gesunden Lebensstils diskutiert. Die Ergebnisse der Diskussion (z. B. hinsichtlich der formalen Gestaltung textbasierter Handreichungen als auch der Bedarfe und Wünsche bezüglich flankierender Themen) wurden in das Materialienportfolio des Bewegungsprogramms von PROMOTE II aufgenommen. Die Dokumentation der partizipativen Entwicklung ist als zusätzliches Online-Material verfügbar [12].

### Evaluation der „Fit im Nordwesten“-Intervention und Materialien

Die Evaluation der entwickelten bzw. adaptierten Materialien erfolgte im Rahmen eines 9‑monatigen randomisierten Feldversuchs zum Vergleich web-basierter und print-basierter Komponenten des Bewegungsprogramms (nähere Informationen zum Studiendesign und randomisierten Gruppen bieten das Studienprotokoll und erste publizierte Ergebnisse [10, 11]). Die wahrgenommene Nützlichkeit der Interventionskomponenten wurde in einem Feedback-Fragebogen mit selbstgenerierten 5‑Punkte-Likert-Skalen von „überhaupt nicht hilfreich“ bis „sehr hilfreich“ erfasst.

## Ergebnisse

### Die „Fit im Nordwesten“-Toolbox

Im Rahmen einer Studie evaluierte print-basierte Materialien wurden als theoriebasierte und praxisrelevante Handlungsempfehlungen aufbereitet und bereitgestellt.

Erste Ergebnisse konnten in der Subgruppe der Personen mit sehr hohen Sitzzeiten zu Beginn der Studie signifikante Steigerungen der körperlichen Aktivität und eine Reduktion der Sitzzeit belegen [14]. Die Evaluation des Feedbacks zum Interventionsmaterial ergab eine hohe Zufriedenheit: Etwa 90 % der Teilnehmer:innen beurteilten das Programm als „etwas hilfreich“ bis „sehr hilfreich“ zum Erreichen der Bewegungsempfehlungen. Detaillierte Informationen sind an anderer Stelle einsehbar [11].

In der Studie mit zwei Projektphasen (über sechs Jahre) konnten Erkenntnisse zur Primärprävention für ein gesundes Altern erlangt und in Form gebrauchsfertiger Instrumente und Arbeitshilfen zur Förderung körperlicher Aktivität zur Verfügung gestellt werden. Die resultierenden Materialien basieren auf der partizipativen Entwicklung eines Interventionskonzepts sowie der Ausarbeitung eines sportwissenschaftlich evidenten, gruppenbasierten, 10-wöchigen Bewegungsprogramms (inklusive altersgerechter Übungskataloge, Bewegungstagebüchern und kommentierten Bewegungsempfehlungen für Kraft, Ausdauer, Beweglichkeit und Gleichgewichtsfähigkeit zur individuellen und selbständigen Nutzung).

Die Erkenntnisse und Materialien stehen aufbereitet für Theorie und Praxis, u. a. als Vorlagen im .pdf- und .ppt-Dateiformat, als „Fit im Nordwesten“-Toolbox zur Verfügung (s. Infobox 1). Die Nutzung der Materialien ist unter einer offenen Lizenz, für die die Nennung der Referenz [12] die einzige Bedingung ist, jederzeit möglich.

#### Infobox 1 Mehr Informationen zum Thema

„Fit im Nordwesten“-Toolbox im Web:

http://www.aequipa.de/materialien/promote.html

Methodik des „World Café“ in der Methodenkartei der Uni Oldenburg:

https://www.methodenkartei.uni-oldenburg.de/uni_methode/world-cafe/

### Inhalt der „Fit im Nordwesten“-Toolbox

**Abb. 3:**
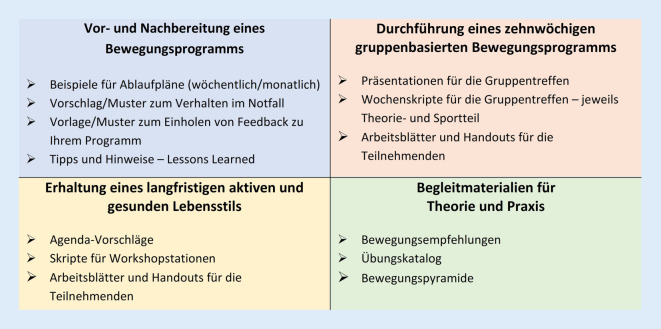
Die Materialien der „Fit im Nordwesten“-Toolbox

Die Toolbox enthält vier thematisch organisierte Materialiensets mit z. T. unterschiedlichen Adressat:innen und Themenfeldern (Abb. 3).

Das Dokumentenset „Materialien für die Vor- und Nachbereitung eines Bewegungsprogramms“ enthält eine Auswahl an Materialien für die Organisation eines angeleiteten Bewegungsprogramms. Darin werden u. a. auch Erkenntnisse geteilt, die sich im Zuge der Studie und Intervention als nützliche Pfeiler in Planung und Umsetzung herausgestellt haben. Basierend auf den Erfahrungen sollten beispielsweise Ablaufpläne, als Handreichung für die Zielgruppe, nicht nur über Ort, Zeit und Veranstaltungstitel informieren, sondern ebenso das konkrete Ziel der Veranstaltung verdeutlichen und inhaltlich kohärent konzipiert sein. Dafür werden Muster bereitgestellt, die sich an den partizipativ entwickelten Themen rund um gesundes Altern (über Bewegung hinaus) orientieren. Das angebotene Tool zum Einholen eines Programm- bzw. Veranstaltungs-Feedbacks kann dazu dienen, die Veranstaltungen zu evaluieren und flexibel anzupassen.

Die „Materialien für die Durchführung eines beispielhaften zehnwöchigen gruppenbasierten Bewegungsprogramms“ richten sich an Akteur:innen, die in die Planung und Durchführung von Bewegungsangeboten involviert sind, und beschreiben exemplarisch die Umsetzung eines zehnwöchigen gruppenbasierten Vor-Ort-Bewegungsprogramms, bestehend aus einer wöchentlichen Sportveranstaltung, inklusive eines jeweils ca. 10- bis 20-minütigen Theorieteils zu einem speziellen Gesundheitsthema (Gesundheitsaufklärung). Basierend auf den Erfahrungen sollten die entsprechenden Theorie- und Sportteile auf das jeweilige Veranstaltungsthema abgestimmt sein, sodass die Theorie von den Angebotsteilnehmer:innen direkt und möglichst alltagstauglich in die Praxis umgesetzt werden kann. Die Toolbox enthält sowohl Skripte mit konkreten Informationen, Übungen und Präsentationsvorschlägen für Gruppenleiter:innen als auch Handouts für die Teilnehmer:innen.

Die „Materialien zur Erhaltung eines langfristigen aktiven und gesunden Lebensstils“ sind für die Begleitung eines 6‑monatigen Programms konzipiert und richten sich speziell an ältere Erwachsene, die vor Kurzem ihr Bewegungsverhalten optimiert haben und dieses zur Gewohnheit machen möchten. Erfahrungsgemäß ist die Gefahr, nach dem Ende regelmäßiger Veranstaltungen wieder in alte Routinen zurückzufallen, groß. Dementsprechend wird in den bereitgestellten Skripten exemplarisch die Durchführung von Workshops zu Motivationstechniken und zum Umgang mit Barrieren für eine Gewohnheitsbildung und langfristig gesundes Verhalten adressiert.

**Abb. 4:**
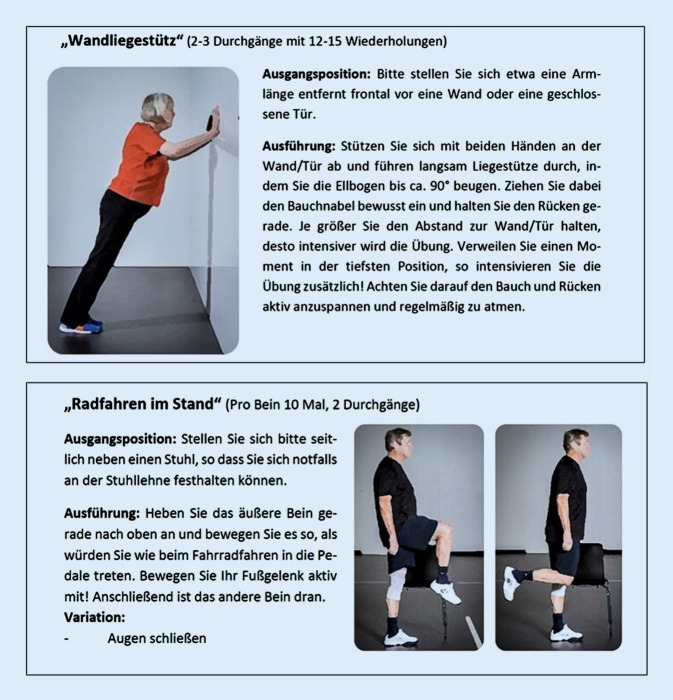
Beispielübungen aus dem Übungskatalog „Fit im Nordwesten“-Toolbox

Abschließend wird eine Auswahl an „Begleitmaterialien für Theorie und Praxis“ zur Verfügung gestellt, die sowohl Anwender:innen als auch Anleiter:innen in der Umsetzung des Programms unterstützen sollen. Die Erkenntnisse aus den Studien beinhalten, dass Empfehlungen zum Ausdauer‑, Kraft‑, Gleichgewichts- und Beweglichkeitstraining nicht nur evidenzbasiert, sondern v. a. für die Teilnehmer:innen nachvollziehbar und umsetzbar sein sollten. Zu diesem Zweck wurden die wissenschaftlichen Hintergründe der Bewegungsempfehlungen zusammengefasst und als übersichtliches, alltagstaugliches Tool angeboten. Alle von Expert:innen, wie Sportwissenschaftler:innen, entwickelten Übungskataloge sollten – über gemeinsames Bewegen in Gruppen hinaus – ein selbstständiges Training der Teilnehmer:innen zuhause fördern (Abb. 4).

Die Materialien können in der Prävention und Gesundheitsförderung zum Einsatz kommen, um ältere Erwachsene zu (mehr) Bewegung zu motivieren.

## Diskussion und Ausblick

Die Materialien der „Fit im Nordwesten“-Toolbox fassen Erkenntnisse aus 6 Jahren Forschung zur Förderung körperlicher Aktivität älterer Erwachsener mit web- und print-basierten Bewegungsprogrammen zusammen. Sie richten sich sowohl an Akteur:innen in unterschiedlichen Anwendungsfeldern und Phasen der Programmplanung und -durchführung, als auch an Personen aus der Zielgruppe in verschiedenen Stadien der Gesundheitsverhaltensänderung.

Verfolgt wird die Vision der Toolbox als „Baukasten“, aus dem individuell benötigte Materialien ausgewählt werden können.

In Zeiten physischer Distanz, u. a. während der COVID-19-Pandemie , sind print- und web-basierte Bewegungsprogramminhalte, die sich für eine individuelle Durchführung zu Hause eignen, essenziell und sehr gefragt [7]. Insbesondere für eine orts- und zeitunabhängige Nutzung sind Hilfsmittel wie Bewegungstagebücher (zum eigenständigen Aktivitätsmonitoring) als auch laientaugliche Übungsanleitungen hoch relevant. Die Bedeutsamkeit dieser individuell nutzbaren Komponenten determiniert sich zusätzlich über die zunehmend eingeschränkte Mobilität im Alter und damit der Möglichkeit zur Bewegung im eigenen Zuhause.

Der Einsatz digitaler Technologien in der Prävention – insbesondere deren Potenziale und Herausforderungen – wird aktuell auch im Rahmen des AEQUIPA-Netzwerks konkret untersucht (Manuskript ist derzeit im Veröffentlichungsprozess). Die Präferenzen für print- bzw. web-basierte Inhalte sowie für spezifische Themen oder organisatorische Gegebenheiten scheinen unter inaktiven älteren Erwachsenen heterogen ausgeprägt zu sein, allerdings war die Akzeptanz der print- bzw. web-basierten Angebote vergleichbar [11]. Die Toolbox kann eine Möglichkeit bieten, Materialien selbstständig und individuell einzusetzen, um den jeweiligen Bedarfen zielgerecht begegnen zu können.

Es ist anzumerken, dass es im Rahmen der PROMOTE-Studie zwar nicht möglich war, die Effektivität einzelner Programmkomponenten objektiv zu messen, dennoch konnten im Rahmen der evaluierten Gesamtintervention insgesamt positive Ergebnisse festgestellt werden. Zudem konnten sowohl das qualitativ erhobene, fragebogenbasierte Teilnehmer:innen-Feedback als auch der Einbezug der Zielgruppe im Rahmen der partizipativen Entwicklungen einen wertvollen Beitrag leisten. Die Effektivität und Eignung der genutzten Materialen über den Zeitraum eines 9‑monatigen Bewegungsprogramms hinaus, ist eine Forschungsfrage, die in zukünftigen Untersuchungen weiter evaluiert werden muss.

## Fazit für die Praxis

- Regelmäßige körperliche Aktivität ist ein wichtiger Bestandteil in der Förderung gesunden Alterns und wird in der Zielgruppe älterer Erwachsener bislang zu wenig umgesetzt. Dies erfordert die Erarbeitung und Dissemination adäquater Konzepte, die die Zielgruppe erreichen können.
- Die zielgruppengerechte Entwicklung und die Dissemination von Methoden und Materialien an die Praxis (z. B. kommunale Anbieter:innen von Sport und Bewegungskursen) sollte bereits frühzeitig in der Projektplanung zukünftiger Forschungsprojekte berücksichtigt werden.
- Die Erkenntnisse und Handlungsempfehlungen sind insbesondere in Zeiten eingeschränkter Mobilität (z. B. aufgrund der COVID-19-Pandemie) aktuell und hochrelevant.
- Evidenzbasierte, partizipativ entwickelte Materialien zur Förderung körperlicher Aktivität stehen in Form einer Toolbox allen Interessierten zur eigenständigen Nutzung bzw. zum Einsatz in eigenen Angeboten zur Verfügung.
